# PRP or not PRP: Is the debate surrounding platelets‐based blood‐derived products evolving?

**DOI:** 10.1002/ksa.12655

**Published:** 2025-03-13

**Authors:** Enrico Ragni, Michela Maria Taiana, Tomislav Čengić, Laura de Girolamo, Marko Ostojić

**Affiliations:** ^1^ IRCCS Ospedale Galeazzi – Sant'Ambrogio Laboratorio di Biotecnologie Applicate all'Ortopedia Milano Italy; ^2^ Geriatric Traumatology and Hip Surgery Division, Traumatology Department ‘Draskoviceva’ University Hospital ‘Sestre Milosrdnice’ Zagreb Croatia; ^3^ Sports Traumatology Division, Traumatology Department ‘Draskoviceva’ University Hospital ‘Sestre Milosrdnice’ Zagreb Croatia; ^4^ Osteon Orthopedics Trauma and Sports Medicine Clinic Mostar Bosnia and Herzegovina; ^5^ European Society of Sports Traumatology, Knee Surgery and Arthroscopy (ESSKA) Basic Science Committee Luxembourg Luxembourg

## Abstract

The increasing interest in biologic treatments for musculoskeletal disorders has led to the advancement of orthobiologics, particularly in non‐operative care through injectable therapies. However, defining these treatments clearly is crucial for proper clinical application. Orthobiologics include biologic substances that enhance healing, such as cell‐based therapies and blood‐derived products. Among these, platelet‐rich plasma (PRP) is widely used, but its classification remains complex due to variations in preparation methods, platelet concentration, leucocyte content, and activation techniques. Strictly, the term ‘PRP’ refers to plasma enriched in platelets relative to baseline blood levels. Yet, scientific debate persists regarding whether platelet count or enrichment is more significant in clinical outcomes. Additionally, leucocyte‐rich and leucocyte‐poor PRPs offer different therapeutic advantages depending on the target tissue, complicating standardization. Similarly, the presence of red blood cells is generally discouraged, given their association with joint inflammation. Beyond the classical ‘PRP’ formulations, alternative blood‐derived products offer distinct biological effects. A standardized classification system is therefore essential for research and clinical application, emphasizing precise documentation of products' characteristics, including platelet count, activation state and bioactive molecule interactions. Understanding these variables and their impact on patient‐specific conditions will refine orthopaedic regenerative strategies and optimize treatment efficacy.

**Abbreviations**
A2M: alpha‐2‐macroglobulin
ACS: autologous conditioned serum
ESSKA: European Society for Sports Traumatology, Knee Surgery and Arthroscopy
LP‐PRP: leucocyte‐poor PRP
LR‐PRP: leucocyte‐rich PRP
MPV: mean platelet volume
OA: osteoarthritis
ORBIT: Orthobiologics Initiative
PRF: platelet‐rich fibrin
PRGF: plasma rich in growth factor
PRP: platelet‐rich plasma
RBC: red blood cell

1

The growing interest in biologic treatments for tissue repair in a range of musculoskeletal disorders has given rise to the discipline of orthobiologics, with the field of non‐operative care having certainly seen a significant ascent in the use of injectable therapeutic alternatives. However, together with sharp patient indications and administration methods, to a greater extent, it is imperative to shed light on the clear definition of ‘what we are dealing with’ and, as a consequence, ‘what we are proposing to patients’.

As a first cornerstone, orthobiologics are biologic substances that promote healing and regeneration, particularly in bones, cartilage, and soft tissues. Orthobiologics leverage the body's natural healing processes to enhance recovery from injuries or surgeries. Currently, available regenerative medicine modalities include a rich portfolio of blood‐derived products, point‐of‐care cell‐based preparations (e.g., bone marrow aspirate concentrate, stromal vascular fraction, microfragmented adipose tissue and amniotic suspension allograft) or good manufacturing practice‐expanded alternatives such as mesenchymal stromal cells (from different autologous or allogenic sources) or chondrocytes. Among these options, the most commonly used conservative treatments are the blood‐derived ones, due to their versatility and performance for different clinical scenarios, as stated by the recent European Society for Sports Traumatology, Knee Surgery and Arthroscopy Orthobiologics Initiative (ESSKA‐ORBIT) [9] and ESSKA‐International Cartilage Regeneration and Joint Preservation Society [7] consensuses. It should be noted that the different blood‐derived products are often generically referred to as platelet‐rich plasma (PRP), which is the proper definition only for a specific subgroup of plasma preparations enriched in platelets (PRP). To add entropy to the system, PRP itself, even if correctly described and proposed to patients, may be produced by various devices, and the procedures may vary in terms of platelet count, presence of red blood cells (RBCs), leucocytes, platelet activation status, and other factors [9]. Thus, a proper definition, alongside clear indications, is imperative.

So, ‘which blood‐derived products can be considered PRP’? At first, the term ‘PRP’ clearly deals with the concept of plasma preparations enriched in platelets with respect to baseline values in patients’ blood. This is both a crucial and a tricky parameter, since baseline blood values in different individuals may affect final platelet enrichment regardless of concentration. Scientists and clinicians have primarily debated the importance of platelet count over platelet enrichment. In this frame, a recent paper suggests how platelet concentration may positively correlate with clinical outcomes in knee OA, with a higher platelet concentration being associated with a lower failure rate and greater improvement compared to PRP with a lower platelet concentration [1]. However, the ESSKA‐ORBIT consensus, while highlighting the importance of both platelet concentration and total platelet count as key determinants, does not currently support a clear correlation between platelet numbers in PRP and clinical response. This remains a subject for further investigation, with future refinements expected based on emerging, cutting‐edge findings [9]. This could be due to platelet responsivity in releasing growth factors, the presence of other bioactive molecules in the plasma, and the patient's unique characteristics, comorbidities, and medications. Therefore, should both low‐(2–3×) and high‐(>5×) enriched or low‐(4–500 × 10^6^ platelets/mL) and high‐(>1000 × 10^6^ platelets/mL) concentrated products be similarly considered ‘PRP’? At present, the answer is: Yes, albeit a mandatory corollary is to clearly describe the devices and procedures to patients and to record the baseline whole blood cellular and platelet composition, as well as those of the produced PRP preparation, improving the understanding of the efficacy to eventually suggest a PRP type over the other for a target pathology. Accordingly, a still underestimated advice is the clear and aware indication of the most adequate PRP bioformulation to treat a specific pathology or condition.

In this frame of quality control measures in clinical research setups, other parameters might be considered and are under investigation by researchers and clinicians to better define a product as ‘PRP’ or to distinguish between different PRP types. In the context of endorsing platelet role, recent research suggested that rather than platelet concentration or fold increase, the mean platelet volume (MPV) may be a useful parameter for studying the storage capacity of platelets, which are the most diverse blood components in terms of size. Higher MPV may indicate a higher concentration of bioactive molecules. Variations in platelet size might affect platelet density, resulting in PRP with comparable platelet concentration but varying MPV and biologic characteristics [11]. Thus, a parameter to define different ‘PRP’ products in the future might be the MPV value, albeit further studies are needed.

A similar approach is under consideration for leucocytes. Although often PRP therapies are mentioned and proposed to patients just as ‘PRP’, leucocyte content sharply parcels clinical products based on the kit/device. Leucocytes, including neutrophils, lymphocytes, and monocytes, perform numerous biological functions that initiate and promote inflammation as a rule of thumb. The benefit of including leucocytes in the PRP product is a topic of contention alongside platelet count. As an example, for cartilage, tendon and muscle damage, some preclinical research suggests that leucocytes may be harmful due to their release of pro‐inflammatory and catabolic mediators like metalloproteinases, and may reduce the overall benefits of PRP. At the same time, other studies recommend their usage due to the release of helpful cytokines, especially when an initial pro‐inflammatory response is needed [12]. Accordingly, in recent years for joint pathologies, there has been a shift towards selecting the most appropriate leucocyte abundance/depleted‐related product to modulate the joint environment, albeit limited clinical data exists on the comparison of leucocyte‐rich (LR) and leucocyte‐poor (LP) PRPs. In a recent double‐blind, randomized trial, Filardo's group showed how LR‐PRP or LP‐PRP injections produced similar clinical improvement at the 12‐month follow‐up in patients with symptomatic knee osteoarthritis (OA) [3]. These results are consistent with the ESSKA‐ORBIT Consensus, which does not support one type of PRP over the other and considers both LP‐PRP and LR‐PRP valid options for the management of knee OA when PRP is considered [9]. Thus, the presence of white blood cells during PRP treatment may be advantageous or harmful, depending on the disease and tissue, and this must be clearly investigated and mentioned to patients when describing the PRP products to be infiltrated.

Being PRP a complex preparation over the abovementioned platelets and leucocytes, the starting question might be renewed from the foundations. This is because, even if largely neglected, RBC presence should also be weighted. Having in mind, for example, that patients with haemophilia develop arthropathy due to repetitive hemarthrosis with hemosiderin deposits in the joint, the presence of erythrocytes in PRP is considered undesirable [14]. Recently, in vitro studies showed that erythrocytes have a negative impact on the intra‐articular environment, resulting in decreased proteoglycan synthesis and increased chondrocyte apoptosis [6]. Consistently, almost all commercial devices deplete RBC from plasma, reducing other degrees of variability between preparations. These characteristics reinforce the notion that PRP should not be viewed as a simple material but rather as a ‘process’ that requires the above‐mentioned parameters to be carefully recorded. In this view, over the past decade, several alternative classification systems for PRP products have been developed, but none have gained popular recognition, despite the efforts of the scientific community in recommending the use of at least one of them when describing the product used. This could be due to a lack of a comprehensive method for PRP preparation and administration, as well as researchers' inability to obtain first‐hand data on PRP product characteristics, particularly when using commercial kits in an outpatient setting. For a definitive classification system, data on PRP procedure, type of disease, and patient features must be matched. This is because responsiveness can be influenced by a variety of factors. Applying this approach from the start of research and clinical use could improve our understanding of the scientific evidence of different PRP products in treating joint diseases like knee OA and other musculoskeletal disorders. A milestone in this scenario is the MIBO approach, a consensus on the minimum reporting requirements for clinical studies evaluating biologics (including PRP) in orthopaedics [10]. However, at present, it is scarcely preferred.

To add a piece of knowledge to this ambitious picture, in 2020, it was produced an outline for the ‘minimum reporting requirements’ separately for in vitro, in vivo and clinical trials together with a PRP novel coding system based on six parameters (N1 to N6) encompassing the abovementioned platelet composition (N1N2) and purity (N3N4), alongside activation (N5N6) [8]. The last scores add another piece to PRP heterogeneity and evolve even further the answer to our question. If PRP is by definition a plasma preparation enriched in platelets, platelets per se do not have any role without activation and release of alpha granules shuttling cytokines and growth factors. Activation may occur naturally once injected by interaction with the target tissues or can be induced before injection by different substances (calcium, thrombin, polyacrylamide beads, etc.). It was shown how different activation methods influence timing and factor release [2]. Therefore, activation changes PRP composition by drastically reducing platelet number and dramatically increasing the presence of soluble factors. An example is the so‐called plasma rich in growth factor (PRGF), which is obtained by CaCl_2_ activation of PRP before injection [5], where the timing of activation may further unsettle the cards, being the release sequence of compounds incomplete and be revised in the future. Thus, can this preparation without or with activated and degranulating platelets still be called PRP, a platelet‐rich product? Again, a clear definition of production protocol and a strict explanation to patients is the way to describe activated PRP, that is, actually, a ‘soluble factors rich plasma’ rather than a classical ‘PRP’.

Under these premises, the blood‐derived portfolio also encompasses those formulations obtained from PRP for specific applications, such as the abovementioned PRGF. This group also lies PRF, platelet‐rich fibrin, which belongs to the new generation of platelet concentrates. During PRF processing, platelets and leucocytes produce cytokines in response to haemostatic events created in the centrifuged tube [4]. PRF injections relieve, for example, arthritic pain, tendinitis or plantar fasciitis but may also be applied directly to the injured cartilage site. Another blood‐derived product enriched in therapeutic molecules is alpha‐2‐macroglobulin (A2M), which is created by passing PRP through specific filters in a sequential manner, extracting some of the smaller molecules, and producing a plasma product rich in A2M. A2M, a serum protease inhibitor, inhibits practically all proteases, including cartilage oligomeric matrix protein‐cleaving proteinases, matrix metalloproteinase‐13 and pro‐inflammatory cytokines (interleukin‐1‐beta and tumour necrosis factor‐alpha) [13]. This product derived from PRP but not directly relying on platelets amount or activity encourages taking a moment to reflect on the initial question ‘which blood‐derived products can be considered PRP?’, keeping in mind that the PRP definition should be addressed strictly to preparations based on platelets, their enrichment and biological action. A perfect example for this consideration is the position of autologous conditioned serum (ACS), a serum isolated from whole blood where exposure of leucocytes to pyrogen‐free surfaces stimulates the release of a set of anti‐inflammatory cytokines to obtain a product similar to PRGF, albeit without the direct involvement of platelets. Nevertheless, ACS is sometimes erroneously proposed to patients as one of the options in the portfolio of PRP formulations, since platelets' amount or role is not the core of its biological activity. Accordingly, A2M and ACS are kept separate from PRP products in the ESSKA consensus [9].

Overall, the scientific understanding of blood‐derived products has shifted from a straightforward focus to a more faceted concept of modulation of the articular environment. Thus, the day‐by‐day higher number of protocols or commercialized devices to process blood laid the foundations for the search for specific ‘autologous regenerative and anti‐inflammatory’ blood‐derived compounds where the initial platelet enrichment concept is a limiting definition. The crucial impact will be on the study of the interactions of bioactive molecules within the joint and the patients' profiling strategy, which identifies factors that impact success or failure. Addressing unresolved issues with these factors is crucial for optimizing future orthopaedic therapies tailored to patients and pathologies.

## AUTHOR CONTRIBUTIONS

**Enrico Ragni**: Conceptualization; methodology; investigation; resources; data curation; writing—original draft preparation; supervision; project administration; funding acquisition. **Michela Maria Taiana**: Conceptualization; methodology; investigation; data curation; writing—original draft preparation. **Tomislav Čengić**: Conceptualization; methodology; investigation; data curation; writing—original draft preparation. **Laura de Girolamo**: Conceptualization; methodology; investigation; data curation; writing—review and editing. **Marko Ostojić**: Conceptualization; methodology; investigation; data curation; writing—original draft preparation.

## CONFLICT OF INTEREST STATEMENT

Enrico Ragni is a member of the ESSKA Basic Science Committee. Laura de Girolamo is Co‐Chair of ESSKA‐ORBIT working group and ESSKA Board Member. Marko Ostojić is Chair of the ESSKA Basic Science Committee and a member of the ESSKA‐ORBIT working group. The remaining authors declare no conflicts of interest.

## ETHICS STATEMENT

The ethics statement is not available.

## Data Availability

Raw data used for drafting the manuscript are available at: https://osf.io/euvnc/?view_only=2cc37cf229c64d48b1f1b6f223ee10d2.
